# Increasing the working hours of nurses and teachers: Evidence from a discrete choice experiment

**DOI:** 10.1371/journal.pone.0337581

**Published:** 2026-01-16

**Authors:** Melline Somers, Tom Stolp, Francesca Burato, Wim Groot, Frits van Merode, Melvin Vooren

**Affiliations:** 1 Research Centre for Education and the Labour Market, Maastricht University, Maastricht, the Netherlands; 2 Maastricht Graduate School of Governance, Maastricht University, Maastricht, the Netherlands; 3 Care and Public Health Research Institute, Maastricht University, Maastricht, the Netherlands; 4 Maastricht UMC+, Maastricht University, Maastricht, the Netherlands,; 5 Department of Educational and Family Studies, Faculty of Behavioral and Movement Sciences, Vrije Universiteit Amsterdam, Amsterdam, the Netherlands; University of Crete, GREECE

## Abstract

The healthcare and education sectors suffer from shortages of nurses and teachers. Extending their working hours has often been proposed as a solution to reduce shortages. In this study, we conduct a discrete choice experiment (DCE) in the Netherlands to elicit nurses’ and teachers’ preferences for different jobs and working conditions. We present both nurses and teachers with nine hypothetical choice sets, each consisting of two jobs that differ in seven observable job attributes. From the DCE, we infer workers’ willingness to pay for these different job characteristics. Moreover, we calculate how many additional hours they would be willing to work if a specific workplace condition were met. We find that both nurses and teachers most negatively value high work pressure. Spending a lot of time on patient-related tasks is highly valued by nurses, followed by having more control over working hours. Next to work pressure, teachers place significant importance on receiving social support from both colleagues and managers. Part-time teachers and nurses require a 23 and 21 percent increase in net hourly wages to accept a full-time working contract, respectively.

## 1 Introduction

Globally, there are significant shortages of workers in both the healthcare and education sectors [1,2]. The shortage of workers in the healthcare sector poses a major challenge to achieving the goal of universal health coverage [3,4]. According to the World Health Organization, the rising demand for health and social care will generate 40 million new jobs by 2030 [5]. Similarly, the global shortage of 69 million teachers required to meet the 2030 educational goals hampers the pursuit of inclusive education [6]. Like the healthcare sector, teacher shortages have been exacerbated by the Covid-19 pandemic [7]. Ensuring the availability and quality of teachers is crucial for the long-term success of students [8].

Although many countries around the world encounter challenges with their healthcare and education workforce [9], the assessment of workforce shortages needs to be appraised within national contexts. The factors determining the demand and supply of nurses and teachers are country- and region specific. For example, the amount of resources allocated to healthcare and education systems, the demographic characteristics of inhabitants, the public image of the nursing and teaching profession, and the bargaining power of nurses differs across contexts [10]. Consequently, countries might require different approaches to address the problem of staff shortages in these sectors.

In many EU counties, such as Belgium, Italy, Germany, and Switzerland, labour shortages in multiple sectors are severe while the labour supply is simultaneously characterised by a large share of part-time workers [11,12,13,14,15]. Perhaps the most prominent examples are the nursing and teaching sectors in the Netherlands, which have a notable prevalence of part-time employment. On average, nurses work 26 hours per week whilst teachers work 29 hours per week (authors’ own data). Additionally, both fields are predominantly female, with nine out of ten nurses and teachers being women [16,17]. Extending working hours as a potential solution to worker shortages has received widespread consideration in the Netherlands [18,19,20,21]. Multiple reports posit that the staff shortages in the education and healthcare sector can be eliminated if part-time (female) workers would increase their weekly hours [18,20,22,23]. For example, Vissers [20] posits that the teacher shortage could be solved by having all teachers currently working one or two days per week take on an additional day of teaching per week.

Incentivizing nurses and teachers to increase working hours will not only enhance the quality and accessibility of healthcare and education but might also benefit employees. While initially designed to facilitate the labour market participation of individuals with caregiving responsibilities [24], part-time employment can also entail unintended and undesirable consequences. A reduction in working hours has been linked to significant income loss [25], occupational downgrading [26], and long-term earning penalties [27]. Increasing the labour force participation of women can strengthen their position in the labour market and contribute to narrowing the gender wage gap.

In this study, we conduct a hypothetical discrete choice experiment among nurses (*N* = 563) and teachers (*N* = 587) in the Netherlands to analyse which factors may motivate these workers to increase their working hours. We designed a discrete choice experiment (DCE) in which respondents are subjected to nine different choice sets. In each choice set, subjects choose between two hypothetical jobs that differ in contractible attributes (i.e., hourly wage increase, number of weekly contract hours) and other job characteristics (control over working hours, work pressure, availability of an education assistant, support from colleagues and supervisors, task content, and travel time). In addition, we collect information on their background characteristics. DCEs have several advantages. Presenting teachers and nurses with hypothetical jobs encourages respondents to make a trade-off between different job characteristic as they would in real-life choices. Through this approach, we can assess the relative importance of different job characteristics. Furthermore, DCEs offer the advantage of incorporating job characteristics that respondents may not currently experience. This allows us to assess the impact of introducing a specific measurement. Because the job characteristics in our DCE are exogenous to the respondent, we can avoid the problem of unobserved confounding factors present in most correlational studies.

To assess nurses’ and teachers’ willingness to increase working hours, we include multiple job attributes that have been introduced in the academic literature as important determinants of their job utility. Workplace-related features such as irregular working hours and limited social support from colleagues negatively affect nurses’ well-being [28,3,29,30]. Additionally, the task design of teaching and nurse jobs has been noted as an important job feature. For example, teachers frequently complain about an increasing amount of administrative duties which decreases their teaching time [31,32]. Finally, higher earnings are recognised as a factor that increases the extensive margin of labour supply and may therefore be effective in increasing working hours in a contractual agreement [33]. Importantly, job earnings also serve as a yard stick to measure willingness-to-pay for different job attributes.

Furthermore, we assess how differences in teacher and nurse characteristics – including gender and care responsibilities – coincide with differences in job attribute preferences. One important determinant of nurses’ and teachers’ labour supply are factors affecting the value of their time in other activities, such as the number and ages of children in the home, and the household’s income from other sources [34,35]. Evidence from the Netherlands also suggests that childcare and household responsibilities are among the most important reasons for nurses and teachers to work part-time [36]; Stichting Het Potentieel [37]. Part-time work is widely used as a flexible working arrangement enabling women, especially mothers, to better allocate time between work and family, thereby avoiding exit from the labour market to perform household tasks [38]. Part-time nurses indeed report lower levels of work-family conflict compared to full-time ones, especially among pre-school children’s mothers [39].

A limited, albeit growing, number of studies use a DCE to test nurses’ and teachers’ job preferences. We contribute to this body of literature in several ways. While most DCEs among nurses have been conducted in developing countries (see, e.g., [40,41,42], our study focusses on a high-income country. Among those studies conducted in high-income countries [43,44,45], only Scott et al. [45] examine how nurses can be incentivised to increase their working hours. Scott et al. [45] find that the labour supply in terms of hours worked among nurses and midwives in Australia is highly inelastic, and their results might not be transferable to other contexts. Several studies have applied DCEs in the context of education. In contrast to earlier studies that focus on pre-service teachers [46], early career teachers [47], and former teachers who left the profession [48], we focus on in-service teachers. Those studies that do focus on in-service teachers do not include working hours as a job attribute [48,49], and thus do not focus on the potential for reducing shortages by motivating workers to increase their weekly hours.

Our findings show that both nurses and teachers prefer low work pressure, valuing it as the most negative job attribute. Nurses require a net hourly wage increase of €3.26 to compensate for high work pressure, while teachers demand €4.06. Furthermore, nurses highly value time for patient-related tasks, while teachers emphasize support from colleagues and managers. Control over working hours is important to both groups. Nurses prefer a 24-hour workweek, while teachers are indifferent between 24 and 32 hours. Nurses require a net wage increase of €2.10 per hour to increase their labour supply from 24 to 32 hours, and teachers needing €1.99 per hour to increase their working hours from 32 to working full-time. However, our heterogeneity analyses show that part-time workers demand a significantly higher wage increase to shift to full-time work.

This paper is structured as follows. In the next section, we review the literature on discrete choice experiments among nurses and teachers, with the aim of measuring their job preferences. In Section 3, we present the design of our experiment and our empirical strategy. In Section 4, we discuss the data and in Section 5, we present our results. In Section 6, we conclude and provide a discussion of our results.

## 2 Related literature

To date, existing DCEs among nurses have largely focussed on developing countries (see, e.g., [40,41,42]. Only a limited number of studies have conducted a discrete choice experiment (DCE) to examine nurses’ job preferences in high-income countries [43,44,45]. Fields et al. [43] investigate the hospital job preferences in hospital medical or surgical units of registered nurses in the US. Their DCE includes eight attributes, namely, earnings, nursing voice in management, tuition reimbursement, scheduling, patient care team, leadership, location, and nursing sensitive patient care outcomes. Nurses mostly valued a cohesive patient care team, readily available and responsive nursing leadership, and a strong nursing voice in management. Nurses are willing to forego $39,357 for a cohesive patient care team, $32,769 for available and responsive leadership, and $31,826 for a strong nursing voice in management.

In contrast to Fields et al. [43] who examine the job preferences among registered nurses, Li et al. (2023) analyse the job preferences of nurse practitioners in the US. Their DCE analyses nurses’ preferences for the professional relationship with a physician, patient panel, billing, professional relationship with other professionals, professional relationship with administration, salary, commute time, and setting. Nurse practitioners mostly value cohesive teams, responsive and supportive administration, and not being supervised by a physician. Nurses need to receive an increase in annual income of $21,780 to forego a very cohesive team, $15,280 for practicing with a not very responsive administration, and $21,450 for being supervised by a physician.

To the best of our knowledge, Scott et al. [45] is the only study applying a DCE to investigate how nurses can be incentivised to extend their working hours. Scott et al. [45] examine nurses’ and midwives preferences for job characteristics in Australia and included the following attributes in their DCE: change in earnings, change in hours worked, type of employer (public or private sector), autonomy, shift type, processes to deal with violence and bullying, and nurse/midwife to patient ratio. Autonomy, working hours, and processes to deal with violence and bullying were valued most highly. Nurses and midwives would be willing to forego 19% of their annual income for adequate autonomy (compared to poor autonomy), and 16% for adequate processes to deal with violence and bullying (compared to poor processes). The findings of Scott et al. [45] show that it is likely to be difficult to encourage nurses and midwifes to work additional hours, as they need to be paid an additional 24% to increase their working hours by 10% ($73 per hour).

A growing literature uses DCEs to quantify the job preferences of teachers, including pre-service teachers [46], early career teachers [47], and former teachers who left the profession [48]. De Cort and De Witte [46] conduct two DCEs to elicit the preferences of pre-service teachers in Flanders. The first DCE consists of a series of job choices with four general working conditions that are not specific to the teaching profession: workload, opportunities for career advancement, frequency of working with young people, and salary. The second DCE consists of a series of choices characterized by working conditions that are specific to the teaching profession: support from school team, difficult student behaviour, and autonomy over the curriculum. Unlike previous studies, De Cort and De Witte [46] also consider pre-service teachers’ perceptions of these working conditions, both within a teaching career and in their preferred alternative career. Pre-service teachers highly value being able to work with young people, equivalent to an 8.1% salary increase, but such opportunities are scarce in alternative careers. They value a low workload and ample career opportunities over being able to frequently work with young people. The average pre-service teacher would prefer a career in which they would earn 10% more over a career in which they are able to frequently work with young people. Few career advancement opportunities is the most important reason for pre-service teachers to opt for their preferred alternative career. Teachers’ lower career advancement opportunities decrease the attractiveness of the teaching profession by the equivalent of an 8.4% salary decrease. High-performing pre-service teachers particularly value career advancement opportunities and curricular autonomy.

Burke et al. [47] assess the preferences for ten different types of support among beginning teachers in Australia in the form of affirmation, resources, collegial opportunities, mentoring, and professional development. They distinguish between teachers with intentions to stay in the profession and those with intentions to leave the profession. Early career teachers with intentions to depart the profession attach relatively more value on the sharing of resources, cooperative teaching and planning, offsite discussions about classroom management and programming with mentors, and having a greater professional voice. Teachers with intentions to stay place greater relative value on observing and discussing teaching practices with more experienced teachers at their school. Burke et al. [47] did not estimate teachers’ willingness to pay for these different types of support.

Johnston [49] examines primary- and secondary school teachers’ preference for fourteen attributes including salary structure, retirement benefits, performance pay, class size, principal support, and time-to-tenure. The most highly valued attribute appears to be having a principal who is supportive with disruptive students. The utility of having a supportive principal is equivalent to a permanent salary increase of $8,670. The presence of a supportive principal also increases teachers’ willingness to teach in disadvantaged schools. Moreover, Johnston (2020) finds that while highly rated teachers mostly have similar preferences as lower rated teachers, highly rated teachers have stronger preferences for schools offering performance pay.

A recent vignette study from the Netherlands analyses which working conditions lower the chance that primary school teachers leave the profession and increase the chance that former primary school teachers re-enter the education sector [48]. The DCE includes a permanent net wage increase, the amount of time that can be spent on tasks other than teaching or preparation, career development opportunities, free childcare, availability of a teaching assistant, and autonomy. For primary school teachers, the strongest effects are found for a wage increase and the availability of a teaching assistant. A 10% wage increase lowers the probability that teachers leave the teaching profession with 4.1%-points. However, a 5% wage increase only lowers this chance with 1.6%-points. The availability of a full-time teaching assistant decreases the probability of leaving with 4.7%-points. The effects of these attributes on the probability that former teachers re-enter the profession are even larger. Cobben et al. [48] estimate that a wage increase of 10% leads to an 8.4%-points increased probability of re-entering the education sector and the availability of a full-time teaching assistant increases this likelihood with 9.8%-points.

## 3 Methodology

### 3.1 Attributes and levels

The aim of a DCE is to assess the trade-off between various attributes, each characterized by specific levels. An attribute in this study is a job-related characteristic, while a level represents a specific value that the attribute can take. One potential weakness of these experiments is that they solely assess the effects of pre-determined attributes, without providing insights into factors not explicitly included in the survey. A condition for the selection of attributes is that they are independent of each other. Given that we can only derive the relative importance of the attributes included in the DCE, it is important to make well-informed decisions about which attributes to include. For guidance in choosing and defining the attributes, we rely on the current literature. In addition, we discussed our selection and formulation of the attributes with several nurses, teachers, and other experts in the field. We restricted the DCE to attributes that are relevant for nurses and teachers, modifiable and feasible for implementation by employers.

For both nurses and teachers, we included the following attributes: a structural net increase in hourly wages, control over working hours, work pressure, support from colleagues and managers, time spent on patient- or teaching related tasks, and contract hours. For nurses, we also included travel time to work as an attribute and for teachers we included the availability of a teaching assistant as an attribute. For all attributes, except for contract hours, we only included two different levels. If we would include more than two levels per attribute, the number of survey questions would increase rapidly, and this would likely have a negative effect on the response rate.

For the first attribute, the net increase in hourly wages, we distinguished between two levels, namely a 1 euro increase and a 3 euro increase. We explained to the respondents that this net hourly wage increase entails a one-time but permanent increase on top of an inflation adjustment equal for all sectors. We emphasized that the wage increase would be on top of any inflation adjustments as wages in many sectors were adjusted for inflation at the time of the survey. Moreover, we indicated that respondents would maintain allowances such as healthcare-, housing-, and childcare allowances, and the child benefits. This is important because most allowances are income dependent and many workers indicate that although they are willing to increase their working hours, they refrain from doing so due to concerns about a reduction in these allowances. To ensure respondents’ understanding of what a 1 euro or 3 euro hourly wage increase entails, we also stated that a 1 euro increase is equivalent to a net monthly increase in earnings of about 160 euro on a full-time contract and that a 3 euro increase equals a monthly increase of about 480 euro. We include this wage attribute in our DCE as is it enables us to express the other job aspects in terms of willingness-to-pay. Moreover, previous research demonstrates that wage increases can positively affect the labour supply among nurses and teachers. This holds for both conditional wage increases (see, e.g., [50,51,52,53,54]) as well as unconditional wage increases (see, e.g., [55,54,56]). Askildsen et al. [55] also show that registered nurses in Norway increase their working hours in response to an exogenous increase in salary.

For the second attribute, control over working hours, we distinguished between “little” and “much” control over working hours. “Little” means that workers have no influence on their working hours and the days they work, while “much” means that they have influence on their working hours and the days they work. Prior research has shown that shift work, including evening and night shifts, can have adverse effects on health and well-being [57,58]. Especially workers with children might have an aversion against evening and night shifts, given the decline in parental acuity [59], along with the potential for diminished behavioural, cognitive, and physical well-being among children, compared to those whose mothers work standard daytime hours [60,61,62,63]. Although teachers do typically not work in shifts, more flexible working hours can also improve teacher wellbeing and increase teacher retention and recruitment [48], and potentially also increase teachers’ willingness to work more hours.

Our third attribute, work pressure, is an important source for work-related stress, emotional exhaustion, burnout symptoms, and the inability to recover [64,65,29]. Consequently, a high work pressure increases the risk of entering or quitting the profession [66,67], and the need to recover from work might prevent workers to increase their working hours. We included two levels for this attribute, namely “normal” and “high”. “Normal” work pressure implies that workers can meet the demand of their job and as a result, they have enough energy and time for activities outside work. In contrast, “high” work pressure means that workers are not able to meet the demands of their job and as a result, they have little time and energy for activities outside work.

For the fourth attribute, support from colleagues and managers, we distinguished between the levels “little” and “much”, little meaning that most of the time workers do not feel supported in their work and the decisions they make. “Much” support implies that most of the time workers feel supported in their work and the decisions they make. Social support from co-workers and supervisors is an important predictor of work engagement, job satisfaction, and preventing burnout, especially when job demands are high [68]. Importantly, social support at work may be particularly important for employees who have high demands in their private life [69]. Hence, social support might alleviate stress, increase workers’ satisfaction, and thereby potentially increase their willingness to extend working hours.

The fifth attribute was formulated differently for nurses and teachers but both related to the task content of their work. For nurses, the levels of this attribute capture what percentage of their time they can spend on tasks related to patient care. Here, we distinguished between 40% and 80% of the time. The remaining time would be spent on tasks not related to patient care. Tasks related to patient care include activities like performing medical procedures and supporting patient and family. Tasks unrelated to patient care include activities such as administration. For teachers, the levels varied between spending 60% and 80% of the time on teaching-related tasks. Teaching-related tasks include teaching, reviewing, and discussing lesson content, while tasks unrelated to teaching include in-service training, parent meetings and administration. We discussed with several nurses and teachers which levels would be realistic. Being able to spend a large amount of time on tasks related to teaching and patient care might improve the perceived task significance of a job, which in turn positively affects work engagement and satisfaction [70,71].

For teachers, we also included the availability of a teaching assistant as an attribute. Here, we varied between the availability of a teaching assistant for 20% or 50% of the time a teacher works. During the time that a teaching assistant is present, the teaching assistant carries out tasks for the teacher. The availability of a teaching assistant can reduce the workload of teachers.

For nurses, we included travel time as an attribute where we distinguished between a travel time to work of 15 minutes or 30 minutes. The nurse and teacher shortages are particularly high in the largest cities in the ^‌^Netherlands [72], while at the same time, these areas also have the largest hidden reserves [73]. The hidden reserve consists of individuals who are qualified to work as a nurse or teacher, but are not working in these professions. One potential explanation for this observation is that densely populated areas, with their high costs of living, also provide many alternative career options. Hence, one strategy for healthcare organizations and schools is to attract nurses and teachers from (adjacent) regions. With this time travel attribute, we aim to examine to what extent workers are willing to travel for their job and how much they need to be compensated for travelling longer distances.

Finally, we included the number of working hours per week as an attribute as this is our outcome measure of interest. Here, we distinguished between four different levels, namely 16 hours, 24 hours, 32 hours and 40 hours. We included four levels because of the potential non-linearity between the willingness to increase working hours and the number of hours that individuals already work. In other words, the average worker might be more willing to increase their working hours if they for example work 24 hours than if they work 32 hours. Before presenting the choice options, we explained the attributes, their levels, and their meanings to the respondents.

### 3.2 Efficient designs

Given the above described attributes and levels, the subsequent step involves constructing choice sets: sets of alternatives. Each choice set comprises a pair of hypothetical jobs, which vary in their attribute levels. Each hypothetical job encompasses seven attributes: six with two levels each and one with four levels. Consequently, there are a total of 2^6 ^× 4 = 256 possible job alternatives. To ensure that our DCE is manageable, we design a D-efficient procedure that minimizes the number of choice sets required to identify all necessary parameters. This design ensures that all possible trade-offs are represented in the survey with minimal redundancy, whilst minimizing the likelihood of choice sets with identical attribute levels. Moreover, this design guarantees that the probabilities for selecting each alternative are relatively equal, thereby avoiding obvious dominant choices.

**Fig 1:**
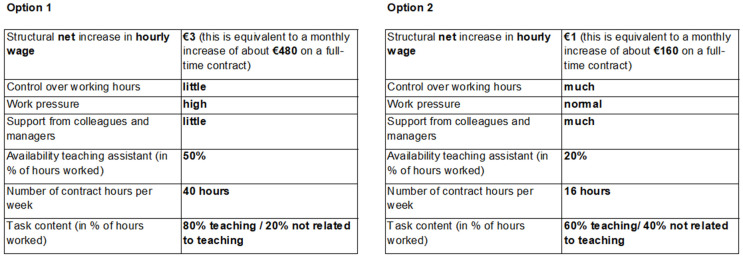
Example survey question.

This approach yields an optimal number of choice sets from the 256 possibilities, with the objective of minimizing the number of choice sets. This results in nine choice sets, each presenting two alternatives. These choice sets are shown in Table 1 for nurses and Table 2 for teachers, with attributes delineated in rows alongside descriptions of their levels. The nine paired choice sets are depicted in the columns. For example, Fig 1 illustrates the first choice set, where a teacher has to make a choice between one of the two described job options. Moreover, we instructed respondents to assume that non-mentioned job characteristics are identical for every job option to limit the risk of omitted variable bias [74]. To minimize the possibility of learning effects, the nine choice sets were randomized in their presentation order.

**Table 1:** Attributes and levels of the nine choice sets for nurses.

	(ii) Choice sets and alternatives (job options A and B)																	
	1		2		3		4		5		6		7		8		9	
	A	B	A	B	A	B	A	B	A	B	A	B	A	B	A	B	A	B
(i) Attributes																		
**1. Hourly net wage increse**	1	0	1	0	1	0	1	0	1	0	1	0	1	0	1	0	0	1
0. 1 euro; 1. 3 euro																		
**2. Control over working hours**	0	1	1	0	1	0	1	0	1	0	0	1	1	0	0	1	1	0
0. little; 1. much																		
**3. Work pressure**	1	0	1	0	1	0	0	1	0	1	0	1	0	1	1	0	1	0
0. normal; 1. high																		
**4. Support from colleagues and managers**	0	1	1	0	0	1	1	0	0	1	1	0	1	0	1	0	1	0
0. little; 1. much																		
**5. Traveltime**	1	0	0	1	1	0	1	0	0	1	0	1	0	1	0	1	0	1
0. 15 minutes; 1. 30 minutes																		
**6. Number of contract hours per week**	3	0	1	2	2	3	3	1	3	2	2	0	2	0	0	1	3	1
0. 16 hours; 1. 24 hours; 2. 32 hours, 3. 40 hours																		
**7. Task content (in % of hours worked)**	1	0	0	1	0	1	0	1	0	1	0	1	1	0	1	0	0	1
0. 60% tasks teaching-related; 1. 80% tasks teaching-related																		

The columns under (ii) depict the nine choice sets in the survey, where participants have to choose between job option A or B. The meaning of the 1s and 0s are is displayed under (i).

**Table 2:** Attributes and levels of the nine choice sets for teachers.

	(ii) Choice sets and alternatives (job options A and B)																	
	1		2		3		4		5		6		7		8		9	
	A	B	A	B	A	B	A	B	A	B	A	B	A	B	A	B	A	B
Attributes																		
**1. Hourly net wage increse**	1	0	1	0	1	0	1	0	1	0	1	0	1	0	1	0	0	1
0. 1 euro; 1. 3 euro																		
**2. Control over working hours**	0	1	1	0	1	0	1	0	1	0	0	1	1	0	0	1	1	0
0. little; 1. much																		
**3. Work pressure**	1	0	1	0	1	0	0	1	0	1	0	1	0	1	1	0	1	0
0. normal; 1. high																		
**4. Support from colleagues and managers**	0	1	1	0	0	1	1	0	0	1	1	0	1	0	1	0	1	0
0. little; 1. much																		
**5. Availability teaching assistant**	1	0	0	1	1	0	1	0	0	1	0	1	0	1	0	1	0	1
0. 20%; 1. 50%																		
**6. Number of contract hours per week**	3	0	1	2	2	3	3	1	3	1	2	0	2	0	0	1	3	1
0. 16 hours; 1. 24 hours; 2. 32 hours, 3. 40 hours																		
**7. Task content (in % of hours worked)**	1	0	0	1	0	1	0	1	0	1	0	1	1	0	1	0	0	1
0. 60% tasks teaching-related; 1. 80% tasks teaching-related																		

The columns under (ii) depict the nine choice sets in the survey, where participants have to choose between job option A or B. The meaning of the 1s and 0s are is displayed under (i).

### 3.3 Econometric approach

To examine how nurses and teachers value different job aspects, we design a discrete choice experiment (DCE). In a Discrete Choice Experiment (DCE), respondents are presented with hypothetical alternatives, each defined by a set of attributes that vary in value. In this study, both nurses and teachers repeatedly choose between two hypothetical jobs that differ in several aspects.

Our methodology in this paper is grounded in rational choice theory which assumes that economic actors seek to maximize their utility. Individual i’s utility of alternative j in choice set s is given by


$$U_{ijs}=V_{ijs}+\in_{ijs}=\beta_{i}^{\prime }a_{jt}+\gamma^{\prime }a_{jt}\cdot z_{i}+\in_{ijs}$$ (1)

where $V_{ijs}$ forms the deterministic part of utility and $\in_{ijs}$ is the unobservable part. The former is written as a linear combination of vectors containing individual-specific preferences $\beta_{i}^{\prime }$ for job attributes $a_{jt}$ which may depend on an observed individual characteristic $z_{i}$ captured by interaction effects $\gamma^{\prime }$. The error term is independently and identically distributed (i.i.d.) by the type-1 extreme value distribution.

As apparent from (1), we allow for individual-specific coefficients, which can be estimated due to the panel structure of the choice experiment. In particular, preference parameters are distributed according to normal multivariate density $\phi (\beta_{i}|\psi )$ where ψ contains a vector μ of preference averages for job attributes and Σ indicates the variance-covariance matrix of preferences. The covariance terms are allowed to vary freely.

The probability that an individual with preferences $\beta_{i}$ selects alternative j instead of k in choice set s is given by


$$P_{ijs}\left(\beta_{i}\right)=Prob\left(U_{ijs}{-U}_{iks}>\text{0}\right)=\frac{exp\left[V_{ijs}(\beta_{i})\right]}{exp\left[V_{ijs}(\beta_{i})\right]+exp\left[V_{iks}(\beta_{i})\right]}=\frac{exp\left[\beta_{i}^{\prime }a_{jt}+\gamma^{\prime }a_{jt}\cdot z_{i}\right]}{exp\left[\beta_{i}^{\prime }a_{jt}+\gamma^{\prime }a_{jt}\cdot z_{i}\right]+exp\left[\beta_{i}^{\prime }a_{kt}+\gamma^{\prime }a_{kt}\cdot z_{i}\right]}$$

which follows from the type-1 extreme value distribution of $\in_{ijs}$. As the aforementioned errors are i.i.d., the probab\ility of observing a particular sequence of individual i’s selection of alternatives in scenarios 1 to S is given by


$$R_{i}\left(\beta_{i}\right)=Prob\left(U_{ij\text{1}}{-U}_{ik\text{1}}>\text{0},U_{il\text{2}}{-U}_{im\text{2}}>\text{0},\ldots ,U_{iqS}{-U}_{irS}>\text{0}\right)=\prod {\mathrm{\backslash nolimits}}_{s=\text{1}}^{S}\frac{exp\left[V_{ijs}(\beta_{i})\right]}{exp\left[V_{ijs}(\beta_{i})\right]+exp\left[V_{iks}(\beta_{i})\right]}$$

The above expression is estimated with the *mixed logit model*. Individual i now makes the sequence of job choices $R_{i}(\psi )$ depending on the distribution parameters of $\beta_{i}$:


$$R_{i}(\psi )=\int \prod {\mathrm{\backslash nolimits}}_{s=\text{1}}^{S}\frac{exp\left[V_{ijs}\left(\beta_{i}\right)\right]}{exp\left[V_{ijs}\left(\beta_{i}\right)\right]+exp\left[V_{iks}\left(\beta_{i}\right)\right]}\phi \left(\beta_{i}|\psi \right)d\beta_{i}$$ (2)

which can be estimated by simulated maximum likelihood techniques (e.g., Revelt and Train, 1998). In particular, the likelihood function $LL(\psi )=\sum_{i=\text{1}}^{N}R_{i}(\psi )$ is maximized with simulated values of $R_{i}(\psi )$ that best fit the data. In the mixed logit estimation we keep the utility of the wage increase fixed to ensure that willingness-to-pay estimates are interpretable (see, e.g., [75]). Standard errors are clustered at the individual level.

Finally, the estimation of (2) allows for unobserved individual heterogeneity in preferences, which is clearly more realistic than assuming $\beta_{i}=\beta$ and contains policy relevant information. Moreover, the mixed logit prevents potential biased estimates of preference parameters [76]. At the same time, the number of parameters to be estimated in the mixed logit model are large which potentially leads to overfitting [75]. Moreover, the estimates of Σ may also capture scale heterogeneity in addition to preference heterogeneity, such that these estimates should be considered with caution. Given the abovementioned (dis)advantages, we present estimates of the mixed logit model – $\beta_{i}\sim \varphi (\mu ,\Sigma )$ – in the main text and compare this to the logit model – $\beta_{i}=\beta$ in the S1 File Online Appendix (S4 Table in S1 File).

## 4 Data

### 4.1 Data collection

The objective of the DCE is to examine how different job aspects affect nurses’ and teachers’ willingness to increase their working hours. The survey and DCE was fielded between 7 July and 16 July 2023 by Kantar Public (henceforth Kantar). Kantar approached respondents from its so-called NIPObase panel. The panel is representative for the Dutch population and the panel members are recruited by Kantar. Hence, individuals cannot select themselves into the NIPObase panel. Survey respondents had provided written consent to Kantar to participate in the research.

At the time of the survey, Kantar did not know which of its panel members were employed as a nurse, but the sector of employment was known. Kantar approached 3,974 of its panel members who were employed in the healthcare sector. Out of the 3,974 invited panel members, 563 respondents (response rate of 15%) completed the survey and DCE. The survey started with a question to check whether respondents were (still) employed as a teacher or nurse. In total, 2,184 respondents dropped out of the survey because they were not part of the target population and 181 respondents did not complete the survey. In December 2022, Kantar conducted a screening among members of the NIPObase panel to assess whether they are employed as a teacher in (special) primary or (special) secondary education. Based on these answers, Kantar has drawn a sample of 902 primary and secondary school teachers who were invited to participate in the survey and DCE. Out of those 902 invited teachers, 587 respondents completed the survey and DCE, resulting in a response rate of 65%. In total, 47 respondents dropped out of the survey because they did not meet the criteria and 55 respondents ended the survey prematurely.

The selected panel members received an email with a link to the survey. Respondents could fill out the survey on different devices such as smartphones, tablets, and laptops. Respondents who participated in the survey and DC experiment received so-called Nippoints that can be exchanged for gift checks or can be donated to charity.

Kantar also provided us with several background characteristics of the respondents including gender, age, household size, and area of residence. In our background survey, we asked additional questions regarding respondents’ family characteristics. These encompassed questions about whether they have children, the age of the youngest child, the presence of a partner, and the number of hours the partner works. With respect to work-related characteristics, we asked respondents about several aspects: the number of years of work experience within their sector of employment, their specific subsector, grades and tracks taught (applicable only to teachers), contract type, gross monthly salary, weekly working hours, and weekly overtime (both paid and unpaid). Additionally, nurses were asked about their commuting time to work.

### 4.2 Sample characteristics and representativeness

Tables S1 and S2 in the S1 File Online Appendix summarize the characteristics of the 563 nurses and 578 teachers who completed the questionnaire. In the sample, 82.9% of nurses and 72.2% of teachers are female. The average age of nurses is just over 48 years, and for teachers, it’s nearly 47 years. Most nurses (24.7%) live in the South, while the largest proportion of teachers (23.3%) live in the East. Most nurses (71.5%) and teachers (71.9%) have a child, with the youngest child’s average age being 17.8 years for nurses and 15.7 years for teachers. The average household size is 2.8 for nurses and 2.9 for teachers, and most have a partner (nurses: 78.2%; teachers: 79.2%). Partners of nurses work 31.1 hours per week on average, while teachers’ partners work 30.8 hours.

In the Netherlands, nurses are trained through either vocational education (mbo) or a university of applied sciences (hbo) programme. In our sample, 47.6% of nurses have mbo training, and 36.9% have hbo training.

Nurses in our sample have an average of 21.7 years of work experience, while teachers average 19.2 years. Most nurses work in hospitals or specialist care (32.5%) or nursing, care, and home care (32.5%). For teachers, 65.6% work in primary education and 34.4% in secondary education. Among secondary teachers, 79.9% teach senior grades, though less than half have the first-degree teaching qualification required for these grades.

Most nurses (87%) and teachers (85%) have permanent contracts. The average hourly salary is €29.72 for nurses and €30.81 for teachers. Nurses work an average of 26.4 hours per week, and teachers 28.7 hours. Both groups report paid overtime (nurses: 4.1 hours; teachers: 1.7 hours) and unpaid overtime (nurses: 1.5 hours; teachers: 5 hours). Nurses’ average commuting time is 23.2 minutes.

For teachers we can analyse the representativeness of the sample by comparing sample estimates to population estimates of six variables. The population estimates originate from the Education Executive Agency (DUO), which maintains administrative records for all teachers in the Netherlands. The population estimates are based on frequencies that are collected on the 1^st^ of October 2022 whereas sample data is generated in July 2023. As both dates fall in the same school year – and teachers typically remain working until the end of the school year – the two sets of estimates arguably yield a valid assessment of representativeness. S3 Table in S1 File depicts the estimates of both samples. In all cases, we only find small discrepancies between sample and population estimates. Group percentages only differ by a few percentage points – e.g., 65.6% percent of the teachers in the sample work in primary education whereas the population-equivalent is 62.5%. Sample means of age, hourly wage and weekly working hours are also similar. Hourly wages constitute the biggest difference of €2.36 which is equal to 0.41 of a standard deviation. Months previous to teachers’ survey responses, wages increased by 10 percent as a result of collective bargaining agreements, however. Consequently, the positive difference between sample and population estimates is arguably biased upwards and becomes smaller if we update the population estimate: 30.31 −28.448 × 1.1 = −€0.48. The similarity between sample and population estimates suggests that preference estimates in this paper signal information about the teacher population at large. Together with Kantar’s statement of collecting data that is representative of the population, the revealed similarity strengthens the notion that nurse data is similarly representative of all nurses working in the Netherlands.

## 5 Results

### 5.1 Average job preferences

Table 3 depicts the preference parameters of the mixed logit model. The first column gives the average sample preferences of the job attributes for nurses and teachers, i.e., the estimated vector of μ of the multivariate normal distribution of $\beta_{i}$. The coefficients all point in the expected direction and are highly significant. In particular, both nurses and teachers prefer more to less of attribute levels that are generally considered favourable. For example, respondents prefer a high to a low wage increase and prefer low work pressure to high work pressure. The preference for working hours per week coincides with the sample characteristics given in Tables S1 and S2 in S1 File. Given the effect on utility of other job attributes, nurses prefer working 24 hours per week the most, which is comparable to their observed working week of 26 hours. They experience greatest disutility by working 40 hours per week. Teachers’ preferences for weekly hours are approximately similar for 24 and 32 hours, which coincides with their sample average of 29 hours. Akin to nurses, teachers value a working week of 40 hours the least. For both nurses and teachers it is clear that working more than 32 hours negatively affects their utility (given their current hourly wage rate).

**Table 3:** Mixed logit estimates of preferences for job attributes.

	Average preferences	WTP (hourly wage)	Heterogeneity (SD)
**NURSES**			
hours per week (baseline: 32 hours)			
16 hours	0.348(0.194)*	€1.18(0.618)*	0.878(0.167)***
24 hours	0.622(0.138)***	€2.10(0.407)***	0.191(0.159)
40 hours	−0.514(0.106)***	-€1.73(0.48)***	0.282(0.19)*
€2 hourly wage increase (fixed)	0.592(0.077)***	€ 2.00	–
flexible working hours	0.547(0.101)***	€1.85(0.445)***	0.682(0.083)***
low work pressure	0.964(0.081)***	€3.26(0.426)***	0.73(0.116)***
high social support	0.467(0.067)***	€1.58(0.327)***	0.306(0.149)*
low travel time	0.025(0.061)	€0.08(0.211)	0.307(0.117)**
more patient time	0.596(0.111)***	€2.01(0.458)***	0.142(0.209)
**TEACHERS**			
hours per week (baseline: 32 hours)			
16 hours	−0.319(0.127)**	-€1.15(0.52)**	1.088(0.252)***
24 hours	0.174(0.101)*	€0.62(0.342)*	0.247(0.17)
40 hours	−0.553(0.091)***	-€1.99(0.39)***	0.182(0.138)
€2 hourly wage increase (fixed)	0.557(0.07)***	€ 2.00	–
flexible working hours	0.434(0.082)***	€1.56(0.312)***	0.325(0.146)*
low work pressure	1.13(0.081)***	€4.06(0.469)***	0.959(0.103)***
high social support	0.446(0.068)***	€1.60(0.323)***	0.196(0.228)
extra teaching assistant	0.281(0.078)***	€1.01(0.303)***	0.121(0.130)
more teaching tasks	0.308(0.089)**	€1.10(0.297)***	0.596(0.165)***

Clustered standard errors are in parentheses. *p < 0.01; **p < 0.05; ***p < 0.01.

The second column of Table 3 depicts teachers’ and nurses’ willingness to pay (WTP) expressed in terms of hourly wages. The WTP is calculated by dividing the preference parameters of non-monetary attributes by the utility coefficient of the €2 wage increase. The estimate is then multiplied by ½ such that WTP is expressed per €1. As mentioned above, working full time generates significant disutility for both nurses and teachers at given wage rates. The results show that hourly wage rates must increase by at least €1.73 and €1.99 to make nurses and teachers willing to work 40 hours as opposed to 32 hours, respectively. Additionally, nurses and teachers are willing to work 32 hours instead of 24 hours if their hourly wage rate is increased by at least €2.10 and €0.62, respectively. In addition to monetary compensation, other job attributes could be offered contractually to incentivise nurses and teachers to work more hours. For example, the estimates suggest that nurses are willing to accept a labour contract in which they work 40 hours as opposed to 32 hours in exchange for more flexible working hours or more patient time. When nurses with a contract of 32 hours are offered flexible working hours, they are willing to increase their working hours by 8.5 hours $\left(\frac{\text{0}.\text{5}\text{4}\text{7}}{-\text{0}.\text{5}\text{1}\text{4}}*-\text{1}*\text{8}\;hours\right)$.

Finally, the last column of Table 3 depicts the estimated standard deviations of the respondents’ preference parameters. In most cases, the estimates are significantly larger than zero yet smaller than the average preference estimates depicted in the first column. This suggests that preference heterogeneity is present, but that the average preference estimate is still meaningful in considering preferences for the sample at large. For instance, approximately 94 percent of the respondents prefer high social support over low social support (keeping other preferences constant). As discussed in Section 4.3, however, we must be cautious in interpreting these heterogeneity estimates as reflecting preference heterogeneity alone, as they may also capture heterogeneity in scale use. For example, individuals may interpret vignettes differently depending on their underlying latent preferences. The sizeable heterogeneity estimates also suggest that the mixed logit model is to be preferred over the logit model in which preferences are assumed to be equal for all individuals. Table S4 in the S1 File Online Appendix documents the estimates of the logit model. Firstly, the model fit is significantly lower than the model fit of the mixed logit in Table 3 ($\chi^{\text{2}}=\text{3}\text{5}\text{6}.\text{9}\text{8},df=\text{4},p<\text{0}.\text{0}\text{1})$. Second, the estimates – both of direct utility parameters and WTP – can differ significantly between both models. The latter may be attributed to the under- and overestimation described in [76]) if heterogeneity in preferences is not taken into consideration.

### 5.2 Individual differences in job preferences

The aim of this paper is to explore which, and to what extent, job attributes might incentivise part-time workers to increase their working hours. As Table 3 reveals considerable preference heterogeneity, this section explores how preferences differ by observed characteristics. Firstly, we consider whether individuals differ in their preferences according to whether they work full-time. Obviously, policy oriented towards increasing working hours can only be geared towards part-time workers and therefore we explore whether their preferences differ. Here, we define working full-time as working more than 32 hours per week. Secondly, we consider two important factors that are closely related to part-time working, namely gender and the caretaking of young children (0–11 year of age).

Table 4 shows the results of the mixed logit model in which the job preferences are simultaneously interacted with the above mentioned worker characteristics. By considering multiple interaction variables in one model, the differences are independent of each other. For example, the differential effect of gender is not driven by working part-time. The first columns shows the preference estimates for the baseline category: Female, part-time workers without young children.

**Table 4:** Mixed logit estimates of job preferences interacted with worker characteristics.

	Baseline	X full-time	X male	X young children
NURSES				
hours per week (baseline: 32 hours)				
16 hours	0.26(0.19)	−1.49(0.51)***	0.56(0.25)**	−0.1(0.42)
24 hours	0.68(0.13)***	−1.18(0.41)***	0.49(0.24)**	−0.47(0.35)
40 hours	−0.77(0.17)***	0.73(0.27)***	−0.02(0.25)	−0.11(0.25)
€2 hourly wage increase (fixed)	0.28(0.09)***	0.11(0.10)	0.06(0.07)	0.08(0.08)
flexible working hours	0.68(0.18)***	0.38(0.60)	0.27(0.24)	−0.19(0.28)
low work pressure	0.99(0.09)***	−0.07(0.23)	0.14(0.20)	−0.19(0.20)
high social support	0.55(0.09)***	0.33(0.78)	−0.16(0.16)	−0.15(0.34)
low travel time	0.13(0.08)*	0.34(0.53)	0.07(0.15)	0.04(0.18)
more patient time	0.92(0.19)***	0.00(0.57)	−0.29(0.21)	−0.15(0.35)
TEACHERS				
hours per week (baseline: 32 hours)				
16 hours	−0.02(0.13)	−0.93(0.18)***	−0.11(0.18)	0.14(0.18)
24 hours	0.50(0.11)***	−0.73(0.15)***	−0.48(0.15)***	0.08(0.15)
40 hours	−0.70(0.10)***	0.61(0.15)***	0.06(0.16)	0.07(0.15)
€2 hourly wage increase (fixed)	0.25(0.07)***	0.20(0.11)*	0.02(0.11)	0.13(0.11)
flexible working hours	0.38(0.07)***	−0.07(0.11)	−0.03(0.10)	0.05(0.1)
low work pressure	1.06(0.09)***	0.02(0.12)	−0.37(0.12)***	−0.05(0.11)
high social support	0.44(0.06)***	0.08(0.10)	−0.33(0.09)***	−0.05(0.09)
extra teaching assistant	0.28(0.07)***	−0.06(0.11)	−0.19(0.10)*	−0.03(0.1)
more teaching tasks	0.33(0.08)***	−0.25(0.13)*	−0.09(0.12)	−0.24(0.11)**

Clustered standard errors are in parentheses. *p < 0.01; **p < 0.05; ***p < 0.01.

The upper panel of Table 4 shows interacted preference estimates for nurses. Nurses’ preferences for working hours depend significantly on actual working hours and gender. In comparison to the baseline category, full-time nurses are approximately indifferent between working 32 hours and 40 hours yet attach negative value to working less than 32 hours. Alternatively, nurses working part-time are strongly dissatisfied by working 40 hours instead of 32 hours and are more satisfied when they work 24 hours as opposed to 32 hours. Hence, it is *more costly* to motivate nurses to work more hours in comparison to the general results depicted in Table 3. For example, enticing a female part-time nurse to work 32 instead of 24 hours requires an hourly wage differential of €4.86 $\left(\frac{\text{2}\cdot \text{0}.\text{6}\text{8}}{\text{0}.\text{2}\text{8}}\right)$ which exceeds the general estimate shown in Table 3 (€2.10). Female part-time nurses require a wage premium of €5.50 to increase working hours to 40 instead of 32 hours. This estimate is equivalent to a 21 percent increase in net hourly wages for nurses. In comparison to female nurses, male part-time nurses attach greater value to working less hours given their current hourly wage. That is, male part-time nurses’ diminished utility of working 32 instead of 24 hours equals 1.17 which indicates that their hourly wage must be raised by €6.88 $\left(\frac{\text{2}\cdot (\text{0}.\text{6}\text{8}+\text{0}.\text{4}\text{9})}{\text{0}.\text{2}\text{8}+\text{0}.\text{0}\text{6}}\right)$ to make them indifferent. Being caretaker of young children does not seem to influence preference for working hours given the influence of other variables in the model. Finally, the interaction variables do not seem to affect preferences for other job attributes significantly even though some sizeable changes are shown.

With respect to teachers’ preferences for working hours, the lower panel of Table 4 depicts similar results in comparison to nurses. Again, full-time female teachers are approximately indifferent between working 32 hours or 40 hours yet negatively value working less than 32 hours. Part-time teachers are dissatisfied by working 40 hours instead of 32 hours and are more satisfied when they work 24 hours as opposed to 32 hours. Female, part-time teachers require an hourly wage premium of €5.60 to increase their working hours from 32 to 40 hours. This is equivalent to a 23 percent increase in average hourly wages of female part-time teachers – i.e., in-sample estimate is €29.74 for female teachers who work between 0 and 32 hours – and shows that working hours are inelastic. In contrast to nurses, the interaction variables now also affect preferences for other job attributes. For example, full-time teachers attach greater value to a wage increase and male teachers gain less utility from low work pressure and high social support. As such, these differences in attribute preferences can be taken into account to entice part-time teachers to work more hours.

## 6 Discussion and conclusion

In many countries, including the Netherlands, the healthcare and education sectors are faced with significant shortages. In the Netherlands, both professions are female-dominated, with a majority of nurses and teachers working part-time. Increasing the working hours of these professionals has often been proposed as a potential solution to alleviate these shortages. We conducted a discrete choice experiment (DCE) where nurses and teachers were presented with a series of trade-offs between two hypothetical job scenarios, each varying in multiple working conditions. This method allowed us to quantitatively assess the relative importance of different job attributes and assign monetary values to individual characteristics of the job roles.

A strong preference for low work pressure is what nurses and teachers have in common. Both among nurses and teachers, high work pressure is valued most negatively. Nurses require a substantial net hourly wage increase of €3.26 to compensate for such work pressure, while teachers demand an even higher compensation of €4.06. Additionally, nurses highly value spending ample time on patient-related tasks, which they equate to a net hourly wage increase of €2.01. Next to work pressure, teachers place significant importance on receiving support from colleagues and managers, valuing this aspect at an equivalent of €1.60 per hour. Furthermore, the ability to control working hours is valued among nurses, who value this attribute at €1.85 per hour. Control over working hours is also valued by teachers, albeit to a lesser extent compared to nurses. Teachers with caregiving responsibilities attach a value of €2.22 per hour to having control over their working hours. The observation that nurses attach more value to this attribute might be explained by the fact that nurses more often work irregular hours.

We also estimated the additional hours nurses and teachers would be willing to work for each attribute introduced into the job. Our findings reveal that nurses prefer working 24 hours per week the most, which is comparable to their observed working week of 26 hours. They experience greatest disutility by working 40 hours per week. Teachers’ preferences for weekly hours are approximately similar for 24 and 32 hours, which aligns with their sample average of 29 hours. Nurses demonstrate a willingness to increase their working hours from 24 to 32 hours with a net hourly wage increase of €2.10, while teachers require an hourly wage increase of €0.62. Hourly wages need to be increased by at least €1.99 to make teachers willing to work 40 hours as opposed to 32 hours.

We also analysed whether job preferences vary by worker characteristics. Our findings show that nurses working part-time have a strong preference for working 24 hours as opposed to working 32 hours. Hence, it is more costly to incentive nurses to work more hours when they have a strong preference to work part-time. For example, motivating a female part-time teacher to work 40 instead of 32 hours requires an hourly wage differential of €5.60, which exceeds the estimate of €2.10 for the full sample and is equivalent to a 23 percent net hourly wage increase For part-time female nurses this amounts to a 21 percent wage increase to be willing to work full-time.

Our study highlights potential interventions that employers can implement to incentivise nurses and teachers to increase their working hours. The success of these interventions will largely depend on the employer’s ability to improve working conditions, such as reducing work pressure and strengthening support from colleagues and managers. Our findings provide valuable insights into how much financial compensation is necessary to offset undesirable job attributes or to reward the presence of positive ones. Crucially, the study also reveals that different groups of workers, depending on their preferences for working hours, may require tailored incentives – such as varying wage increases – to motivate them to take on more hours. The hourly wage premia demanded by part-time teachers and nurses to work full-time suggests that a wage increase conditioned on full-time employment is infeasible.

## Supporting information

S1 File Online appendix. (DOCX)
