# Supplementary material for: Increasing the working hours of nurses and teachers: Evidence from a discrete choice experiment
Source: PLoS One. 2026 Jan 16;21(1):e0337581. doi: 10.1371/journal.pone.0337581 (PMC12810815; doi:10.1371/journal.pone.0337581)
Supplement: S1 File — (DOCX) [file pone.0337581.s001.docx]

**Supporting information**

**Table S1: Summary statistics – Nurses**

|  | N | Mean | SD | Min | Max |
| --- | --- | --- | --- | --- | --- |
| ***Background variables*** |  |  |  |  |  |
| Female | 563 | 0.829 |  | 0 | 1 |
| Age | 563 | 48.307 | 11.520 | 19 | 85 |
| *Area of living* |  |  |  |  |  |
| Big city | 563 | 0.060 |  | 0 | 1 |
| Peripheral municipalities | 563 | 0.037 |  | 0 | 1 |
| West | 563 | 0.290 |  | 0 | 1 |
| North | 563 | 0.123 |  | 0 | 1 |
| South | 563 | 0.247 |  | 0 | 1 |
| East | 563 | 0.243 |  | 0 | 1 |
| *Family characteristics* |  |  |  |  |  |
| Having children | 563 | 0.716 |  | 0 | 1 |
| Age of the youngest child | 403 | 17.772 | 10.157 | 0 | 44 |
| Household size | 563 | 2.845 | 1.399 | 1 | 7 |
| Having a partner | 563 | 0.782 |  | 0 | 1 |
| Weekly hours worked by the partner | 440 | 31.148 | 13.717 | 0 | 70 |
| *Education* |  |  |  |  |  |
| Mbo nursing diploma | 563 | 0.476 |  | 0 | 1 |
| Hbo nursing diploma | 563 | 0.369 |  | 0 | 1 |
| Other | 563 | 0.155 |  | 0 | 1 |
| ***Work-related characteristics*** |  |  |  |  |  |
| Years of experience as nurse | 563 | 21.742 | 13.238 | 0 | 51 |
| *Healthcare sector of employment* |  |  |  |  |  |
| University medical centers | 563 | 0.057 |  | 0 | 1 |
| Hospitals and other specialist medical care | 563 | 0.325 |  | 0 | 1 |
| Mental healthcare | 563 | 0.112 |  | 0 | 1 |
| General practitioners and health centers | 563 | 0.030 |  | 0 | 1 |
| Nursing, care and home care | 563 | 0.325 |  | 0 | 1 |
| Disability care | 563 | 0.117 |  | 0 | 1 |
| Other  *Type of contract* | 563 | 0.034 |  | 0 | 1 |
| *Type of contract* |  |  |  |  |  |
| Permanent contract at a healthcare organization | 563 | 0.870 |  | 0 | 1 |
| Temporary contract at a healthcare organization | 563 | 0.062 |  | 0 | 1 |
| On-call contract at a healthcare organization | 563 | 0.021 |  | 0 | 1 |
| Temporary employment contract | 563 | 0.002 |  | 0 | 1 |
| Secondment contract | 563 | 0.007 |  | 0 | 1 |
| Self-employed | 563 | 0.020 |  | 0 | 1 |
| Other | 563 | 0.018 |  | 0 | 1 |
| *Other work-related characteristics* |  |  |  |  |  |
| Hourly gross wage | 400 | 29.717 | 25.375 | 0 | 33000 |
| Weekly working hours | 563 | 26.433 |  | 0 | 40 |
| Paid hours worked overtime | 563 | 4.144 |  | 0 | 40 |
| Unpaid hours worked overtime | 563 | 1.453 |  | 0 | 20 |
| Travel time in minutes | 563 | 23.231 | 14.857 | 0 | 150 |

**Table S2: Summary statistics – Teachers**

|  | N | Mean | SD | Min | Max |
| --- | --- | --- | --- | --- | --- |
| ***Background variables*** |  |  |  |  |  |
| Female | 587 | 0.722 |  | 0 | 1 |
| Age | 587 | 46.988 | 11.588 | 19 | 80 |
| *Area of living* |  |  |  |  |  |
| Big city | 587 | 0.058 |  | 0 | 1 |
| Peripheral municipalities | 587 | 0.070 |  | 0 | 1 |
| West | 587 | 0.330 |  | 0 | 1 |
| North | 587 | 0.106 |  | 0 | 1 |
| East | 587 | 0.233 |  | 0 | 1 |
| South | 587 | 0.203 |  | 0 | 1 |
| *Family characteristics* |  |  |  |  |  |
| Having children | 587 | 0.719 |  | 0 | 1 |
| Age of the youngest child | 422 | 15.680 | 11.070 | 0 | 56 |
| Household size | 587 | 2.879 | 1.411 | 1 | 8 |
| Having a partner | 587 | 0.792 |  | 0 | 1 |
| Weekly hours worked by the partner | 465 | 30.785 | 14.339 | 0 | 75 |
| *Education* |  |  |  |  |  |
| First-degree teacher | 202 | 0.465 |  | 0 | 1 |
| Second-degree teacher | 202 | 0.441 |  | 0 | 1 |
| Other | 202 | 0.094 |  | 0 | 1 |
| ***Work-related characteristics*** |  |  |  |  |  |
| Years of experience as teacher | 587 | 19.225 | 11.376 | 0 | 52 |
| *Education sector of employment* |  |  |  |  |  |
| Primary education | 587 | 0.656 |  | 0 | 1 |
| Secondary education | 587 | 0.344 |  | 0 | 1 |
| *Primary education teachers: teaching grade* |  |  |  |  |  |
| 1^st^ grade | 385 | 0.270 |  | 0 | 1 |
| 2^nd^ grade | 385 | 0.262 |  | 0 | 1 |
| 3^rd^ grade | 385 | 0.273 |  | 0 | 1 |
| 4^th^ grade | 385 | 0.247 |  | 0 | 1 |
| 5^th^ grade | 385 | 0.265 |  | 0 | 1 |
| 6^th^ grade | 385 | 0.281 |  | 0 | 1 |
| 7^th^ grade | 385 | 0.262 |  | 0 | 1 |
| 8^th^ grade | 385 | 0.278 |  | 0 | 1 |
| *Secondary education teachers: teaching grade* |  |  |  |  |  |
| Junior | 202 | 0.203 |  | 0 | 1 |
| Senior | 202 | 0.233 |  | 0 | 1 |
| Both | 202 | 0.564 |  | 0 | 1 |
| *Secondary education teachers: teaching track* |  |  |  |  |  |
| Vmbo (pre-vocational education) | 202 | 0.564 |  | 0 | 1 |
| Havo (general secondary education) | 202 | 0.604 |  | 0 | 1 |
| Vwo (pre-university education) | 202 | 0.619 |  | 0 | 1 |
| *Type of contract* |  |  |  |  |  |
| Permanent contract at a school/board | 587 | 0.855 |  | 0 | 1 |
| Temporary contract at a school/board | 587 | 0.097 |  | 0 | 1 |
| On-call contract at a school/board | 587 | 0.017 |  | 0 | 1 |
| Temporary contract | 587 | 0.005 |  | 0 | 1 |
| Secondment contract | 587 | 0.003 |  | 0 | 1 |
| Self-employed | 587 | 0.012 |  | 0 | 1 |
| Other | 587 | 0.010 |  | 0 | 1 |
| *Other employment conditions* |  |  |  |  |  |
| Hourly gross wage | 473 | 30.808 | 5.745 | 18.756 | 45.16 |
| Weekly working hours | 587 | 28.656 | 9.128 | 0 | 40 |
| Paid hours worked overtime | 587 | 1.698 | 5.845 | 0 | 40 |
| Unpaid hours worked overtime | 587 | 4.983 | 4.632 | 0 | 30 |

**Table S3: Comparison to population estimates - Teachers**

|  | Sample | | Population | |
| --- | --- | --- | --- | --- |
|  | N | Mean | N | Mean |
| ***Background variables*** |  |  |  |  |
| Female | 587 | 0.722 | 205496 | 0.765 |
| Age | 587 | 46.988 | 205496 | 43.649 |
|  |  |  |  |  |
| ***Work-related characteristics*** |  |  |  |  |
| Primary education | 587 | 0.656 | 205496 | 0.625 |
| Secondary education | 587 | 0.344 | 205496 | 0.375 |
|  |  |  |  |  |
| Permanent contract at a school/board | 587 | 0.855 | 205496 | 0.824 |
|  |  |  |  |  |
| *Other employment conditions* |  |  |  |  |
| Hourly wage | 473 | 30.808 | 205496 | 28.448 |
| Weekly working hours | 587 | 28.656 | 205496 | 29.993 |

**Table S4: Logit estimates of preferences for job attributes**

|  |  | average preferences |  | WTP (hourly wage) |
| --- | --- | --- | --- | --- |
| **NURSES** | |  |  |  |
|  | €2 hourly wage increase (fixed) | 0.279(0.029)*** |  | €2.00 |
|  | flexible working hours | 0.778(0.075)*** |  | €2.78(0.378)*** |
|  | low work pressure | 0.961(0.063)*** |  | -€3.43(0.417)*** |
|  | high social support | 0.575(0.055)*** |  | €2.06(0.287)*** |
|  | low travel time | 0.176(0.050)*** |  | -€0.63(0.185)*** |
|  | more patient time | 0.867(0.091)*** |  | €3.10(0.429)*** |
|  |  |  |  |  |
|  | hours per week (baseline: 32 hours) |  |  |  |
|  | 16 hours | -0.109(0.073) |  | -€0.39(0.263) |
|  | 24 hours | 0.369(0.064)*** |  | €1.32(0.269)*** |
|  | 40 hours | -0.551(0.059)*** |  | -€1.97(0.290)*** |
|  |  |  |  |  |
| **TEACHERS** | |  |  |  |
|  | €2 hourly wage increase (fixed) | 0.466(0.065)*** |  | €2.00 |
|  | flexible working hours | 0.513(0.061)*** |  | €2.20(0.405)*** |
|  | low work pressure | 1.293(0.072)*** |  | -€5.55(0.860)*** |
|  | high social support | 0.515(0.057)*** |  | €2.21(0.395)*** |
|  | extra teaching assistant | 0.343(0.050) |  | €1.47(0.305)*** |
|  | more teaching tasks | 0.251(0.073)*** |  | €1.08(0.345)*** |
|  |  |  |  |  |
|  | hours per week (baseline: 32 hours) |  |  |  |
|  | 16 hours | -0.413(0.076)*** |  | -€1.77(0.422)*** |
|  | 24 hours | 0.159(0.065)** |  | €0.683(0.292)** |
|  | 40 hours | -0.553(0.059)*** |  | -€2.42(0.438)*** |

Clustered standard errors are in parentheses. *p<0.01; **p<0.05; ***p<0.01.
